# Antimicrobial resistance in fecal *Escherichia coli* from different pig production systems

**DOI:** 10.5713/ab.21.0232

**Published:** 2021-08-25

**Authors:** Jamlong Mitchaothai, Kanokrat Srikijkasemwat

**Affiliations:** 1 Department of Animal Production Technology and Fisheries, Faculty of Agricultural Technology, King Mongkut’s Institute of Technology Ladkrabang, Bangkok 10520, Thailand

**Keywords:** Antibiotic Resistance, Deep Litter Housing, *Escherichia coli*, Pig, Systems

## Abstract

**Objective:**

The objective of the current study was to investigate the influences of conventional (CO) and deep litter (DE) systems on antimicrobial resistance in fecal *Escherichia coli* (*E. coli*).

**Methods:**

A cross-sectional study was carried out to detect antimicrobial resistance to *E. coli* in swine fecal samples in CO and DE systems located in western and northeastern Thailand. Individual rectal swab samples were taken only from healthy pigs. A total of 215 individual and healthy pigs were randomly selected for isolation and antimicrobial susceptibility test of *E. coli* by the disc diffusion method. The test panel included amoxicillin (AMX), colistin, doxycycline (DOX), enrofloxacin, gentamicin (GEN), kanamycin, neomycin (NEO), and trimethoprim-sulfamethoxazole (SXT).

**Results:**

There were significant (p<0.05) lower resistance levels for GEN, NEO, and SXT in the DE farms compared to those in the CO farms. There was a lower number of antimicrobial resistance agents (p<0.001) in the DE farms compared to those in the CO farms. This result was consistent with those in western (p<0.01) and northeastern (p<0.01) Thailand. Overall, antibiograms of AMX-SXT and AMX-DOX-SXT were found in the CO (19.09% and 20.91%, respectively) and the DE (16.19% and 24.76%, respectively) farms. No antimicrobial resistance (5.71%) was found and AMX (13.33%) resistant pigs in the DE farms, whereas the pattern of AMX-GEN-SXT (6.36%) and AMX-DOX-GEN-SXT (11.82%) resistant pigs was found in the CO farms.

**Conclusion:**

The DE system for pig farming was superior to conventional pig farming by lowering the resistance level of fecal *E. coli* to GEN, NEO, and SXT, with decreasing the number of antimicrobial resistance agents and inducing a small proportion of pigs to be free from antimicrobial resistance.

## INTRODUCTION

Currently, antimicrobial resistance is an emerging concern for the livestock production sector and also a major concern for human health. Overuse of antimicrobial agents in livestock in Southeast Asian countries is one likely driver of the high antimicrobial resistance [1] as the result of generating antimicrobial resistance reservoirs. One important strategy to reduce antimicrobial resistance is rational antimicrobial use. In Thailand, the national strategic plan has been assigned and aimed to achieve a 30% reduction in antimicrobial use in animals by the year 2021. Consequently, Thai farmers are required to improve antimicrobial use for pig production, including lowering the cost of interventions to ensure widespread uptake in the livestock sector [2]. Increasing disease pressure is a possible explanation for the higher use of antimicrobials in feed for medium-scale pig farms when compared with small-scale pig farms [3]. For large-scale or industrial pig farms, disease pressure was reduced by better management procedures in these farms [4] and high safety level from farm biosecurity, resulting in no antimicrobial use in some pig herds [5]. Higher the limit of stock density for pigs has been proven to lower production performance and increase the risk of getting infections among pigs through altering immune systems [4].

More than 80% of pigs farming systems are contract farming between the primary producers and the agribusiness companies in Thailand [6]. Small-scale pig farming seems to be possible to get less disease pressure as free from constraints of contract farming and lower stock pig density. From an earlier report in northeastern Thailand [7], they found that small-scale pig farms rarely used antibiotics for disease prevention, but mainly for the treatment of diseases without veterinary advice and service when compared with medium-scale pig farms. However, many small-scale pig farmers have struggled with higher feed costs and lower levels of farm biosecurity, resulting in a dramatic decrease in the number of small-scale pig farms in Thailand. This issue could be illustrated by a study in Thailand [8] as increasing numbers of pigs per owner over time but decreasing numbers of pig farm owners. The report [8] also mentioned that smaller-scale pig producers are distributed in more rural regions, so optimal environmental, health, and economic impacts should be concerned. Deep litter farming for pigs should be a solution for small-scale farming with sustainability. The study under tropical conditions [9] indicates that the deep-bedding of coffee and rice husks provided the best choice for economic feasibility in terms of farmer and growing-finishing pigs.

The construction of facilities for deep litter pig keeping is up to 40% cheaper than for conventional facilities and possesses many advantages including a positive effect on pig welfare and health as well as reduced disease prevalence [10]. The fermented deep litter system for growing-finishing pigs could reduce the NH_3_ and odor concentrations as well as NH_3_ emissions [11]. This finding would support the report, which showed deep litter housing with fermented feeding might be an effective technology in reducing occurrences of diseases and increasing the immunity of pigs [12], including a lower prevalence of osteochondrosis [13]. For the deep litter farming situation in Thailand, there are some changes from the past in which farmers operated backyard pigs by using native or crossbred native breeds with ≤5 pigs per owner, small pig holders. Nowadays, many pig farmers applied deep litter system for housing pigs with more commercial purposes by changing to use crossbred commercial breeds (Landrace× Large White×Duroc), larger farm size as community enterprise with their farm parent stocks and serving as natural or food safety pork for consumer needs in the current era. In Thailand, many pig farmers operate the deep litter system without antimicrobial use in their farms because fewer health problems occurred among pigs, resulting in beneficial effects for consumers and sustainability for the farmers. No antimicrobial use in the deep litter system would be a crucial way to reduce antimicrobial resistance for pig production and thus, a possible solution to reduce antimicrobial resistance reservoirs in the pig production industry. However, there are still questions about food safety related to antimicrobial resistance, which may be produced from pig production with the deep litter system. To clarify the issue of antimicrobial resistance among pigs reared in the deep litter system, this study aimed to investigate the influences of conventional (CO) and deep litter (DE) systems on antimicrobial resistance by using samples of fecal *Escherichia coli* (*E. coli*).

## MATERIALS AND METHODS

### Animal care

This study was conducted on four pig farms in Thailand following the guidelines in “The Ethical Principles and Guidelines for the Use of Animals for Scientific Purposes”, edited by the National Research Council of Thailand. The study was approved by the Animal Care and Use Committee, King Mongkut’s Institute of Technology Ladkrabang (Approval number: ACUC-KMITL-RES/2019/005).

### Farm selection and study design

This study was performed from March 2019 to April 2020. A quasi-experiment was designed for the current study by selecting representative experimental pig farms. Key general criteria for including pig farms in this study were no major or important disease outbreaks during the past 2 years and limited antimicrobial use or no antimicrobial use applied for at least 3 years before the start of this study. There were 3 main criteria to select farms for the current study, consisting of i) crossbred (Landrace×Large White×Duroc) used for production, ii) facility of total breeding stock of ≤20 sows and 21 to 50 sows for the CO and the DE system, respectively and iii) no and limited antimicrobial used in pig farm for the DE and the CO system, respectively. There were only two regions in Thailand, western and northeastern, that met those 3 criteria because of producing with native and local pig breeds, and/or an inadequately number of sow breeding stock in other regions (northern, eastern, and southern Thailand). For the third criteria of antimicrobial use, there were no farms available for no antimicrobial used in a small-scale pig farm with the CO system. Thus, the CO farm with limited antimicrobial use was included in the current study, in agreement with the real situation of pig production in Thailand, as mentioned in the introduction part. A representative farm for the CO and the DE system was selected from each region of western and northeastern Thailand. Thus, two small-scale conventional pig farms and two medium-scale deep litter pig farms were enrolled in this study. A cross-sectional sampling to detect antimicrobial resistance to *E. coli* in swine fecal samples in two types of production systems with different regions of farm locations in Thailand is described in Table 1. The two CO farms were classified as limited antimicrobial use farms, while the two DE farms were classified as no antimicrobial use farms. A total of 215 healthy pigs were randomly selected for sample collection according to the proportion of pig age structure of sow, nursery, starter, grower, and finisher (excluding suckling pigs) in each farm.

### Fecal samples collection

Individual rectal swab samples were only taken from healthy pigs by a veterinarian and farmworker assistants. There was a total of 60, 50, 55, and 50 rectal swab samples individually collected from pigs in the CO farms located in western and northeastern Thailand, as well as in the DE farms in western and northeastern Thailand, respectively. The rectal swabs were collected and transported in a transport medium, after which the specimens were transported to the laboratory under a cold chain. They were stored for a maximum of 24 h at 2°C to 4°C until subsequent analysis.

### Isolation and antimicrobial susceptibility test of *Escherichia coli*

The methods of the International Organization for Standardization (ISO 9308–1, 2014) were applied for *E. coli* culture. From each positive sample, three isolates of *E. coli* were kept in skim milk at −20°C for further study. The Kirby–Bauer method (disc diffusion method) was applied to conduct susceptibility testing of *E. coli* isolates to a panel of antimicrobial agents. The antimicrobials in the test panel were chosen based on common use for pigs in the past and the present for both studied regions and represented for different classes of antimicrobials. The test panel included amoxicillin (AMX 10 μg), colistin (COL 10 μg), doxycycline (DOX 30 μg), enrofloxacin (ENR 5 μg), gentamicin (GEN 10 μg), kanamycin (KAN 30 μg), neomycin (NEO 30 μg), and trimethoprim-sulfamethoxazole (SXT 25 μg). *E. coli* ATCC 25922 was used as the quality control strains. Antimicrobial resistance breakpoints were interpreted following the Clinical and Laboratory Standards Institute criteria [14].

### Statistical analysis

Data on antimicrobial resistance were described in percentages. Proportions of antimicrobial resistance between the CO and DE systems were performed by Fisher’s Exact Chi-square tests in SPSS 26.0 (IBM Corp. IBM SPSS Statistics for Windows, Version 26.0 IBM Corp., Armonk, NY, USA). Significant levels were defined at p<0.05. For detecting differences in the number of antimicrobial-resistant agents, the non-parametric test was used for analysis by Mann-Whitney U test for detecting the difference between the CO and DE systems, and by Krukal-Wallis test for detecting differences among age classes in each farm in the SPSS program, as mentioned earlier.

## RESULTS

### Antimicrobial resistance in *Escherichia coli* isolates

From a total of 215 fecal samples, all (100%) were isolated successfully. All *E. coli* isolates were tested for susceptibility to eight antimicrobial agents. Percentages of antimicrobial resistance of overall isolates ranged from a low resistant level at 0% for ENR and COL to a very high resistant level as 98.18% for AMX, even 100% for AMX in the CO farm (Table 2). For overall *E. coli* isolates, there was no difference (p>0.05) between the CO and the DE systems for percentages of antimicrobial resistance of COL, DOX, ENR, and KAN. There was a trend (p = 0.0760) of the lower antimicrobial-resistant level of AMX in the DE farm and a significant (p<0.05) lower antimicrobial-resistant level of GEN, NEO, and SXT in the DE farm when compared to those in the CO farms. For the farms in the northeastern region, there was consistency in antimicrobial resistance with those in the overall *E. coli* isolates, except for no difference (p>0.05) of AMX and SXT resistant level. There was also consistency of antimicrobial resistance level difference of *E. coli* isolates between the farms in the western region, except for no difference (p>0.05) of NEO resistant level.

When the number of antimicrobial resistance agents was considered (Table 3), the results from a quantitative analysis showed a lower number of antimicrobial resistance agents (p<0.001) in the DE farm compared to the CO farm. This result was consistent with those in the western (p<0.01) and northeastern (p<0.01) regions. Minimum, median, and maximum for the number of antimicrobial resistance agents in the CO farm were 1, 3, and 7, respectively, while those in the DE farm were 0, 2, and 6, respectively, for overall *E. coli* isolates. A similar trend was also found in both the CO and the DE farms in the western and northeastern regions. When pig age class was considered for influence on the number of antimicrobial resistance agents, it was rather obvious that the difference in the number of antimicrobial resistance agents between the pigs in the CO and the DE farms resulted from the grower and finisher pigs (Table 3). The highest number of antimicrobial resistance agents was found in the age class of nursery and starter pigs with significant highest (p<0.05) of that in the CO farm from the northeastern region. In the meantime, there was no difference (p>0.05) in the number of antimicrobial resistance agents between the pigs in the age class of nursery and starter, and sow, which were reared in four representative pig farms.

### Antimicrobial resistance profiles and distribution

Patterns of antibiotic resistance (antibiograms) comprised 29 patterns (not show data) observed in this study. From these 29 patterns, the key antimicrobial resistance in this study was AMX, as found in 27 patterns (out of a total of 29). A total of 26 patterns were classified as multidrug-resistant (MDR) according to the definition of “resistant to more than one antimicrobial agent” [15]. There was no antimicrobial resistance found (5.71%) in *E. coli* isolates from the DE farms (Table 4). Overall, antibiograms of AMX-SXT and AMX-DOX-SXT were found in the CO (19.09% and 20.91%, respectively) and DE (16.19% and 24.76%, respectively) farms for approximately 40% to 41% of studied pigs, implying common patterns of antimicrobial resistance for both CO and DE systems in the current study. Approximately 18% of all experimental pigs in the CO farms had antibiograms of AMX-GEN-SXT (6.36%) and AMX-DOX-GEN-SXT (11.82%) resistant to *E. coli*. Approximately 19% of all studied pigs in the DE farms had no antimicrobial resistance (5.71%) and only AMX (13.33%) were resistant to *E. coli*. There was also a major difference in the proportion of antibiogram between the pigs in western and northeastern Thailand. The pigs in the western region had a larger proportion of antibiogram patterns 1, 2, 3, 4, and 6, while smaller proportion for antibiogram patterns 5 and 7 when compared with those in the northeastern region (Table 4).

## DISCUSSION

In the present study, antimicrobial resistance in *E. coli* was investigated from fecal samples of pigs reared in two production systems, namely the CO and DE systems, and two different regions in western and northeastern Thailand. There is still complexity and challenges for antimicrobial resistance because the mechanism of dissemination and maintenance of antimicrobial resistance has not yet been elucidated fully [16]. Thus, the current study aimed to control many factors which could affect the results in the current study, as partly described in the section on the Farm selection and study design. The result is only one representative farm for each pig production system in each region. Important general management and environmental factors were controlled by the process of farm selection, such as farm size, as mentioned in Table 1, opened barn operating system, medium level of farm biosecurity, the stock density of 1.5 to 2.0 m^2^/pigs for finishing barn and disease status by without major disease outbreak. These control criteria led this study to be matched with a quasi-experiment, in which few differences of management in studied farm systems were classified as management specific to the farm system, such as floor type and management and nursery pig grouping after weaning. Hence, the results obtained from the current study would mainly be influenced by the production system. The disc diffusion method was carried out to detect antimicrobial resistance. The high antimicrobial-resistant level in this study was AMX (comparable to ampicillin and penicillin), DOX (comparable to tetracycline) and SXT following earlier reports [3,5,17]. This agreement with earlier studies might be because these earlier reports were conducted during a recent period for starting a campaign for reducing antimicrobial use in pig production in Thailand. When the antimicrobial-resistant level in the CO farm was compared to that in the DE farm, the level of GEN (aminoglycoside agent) resistance was markedly lowered in the DE farms, both in the western and the northeastern regions. In the meantime, the resistance levels of KAN and NEO, which are classified as aminoglycoside agents, might be influenced by the region of the current study and low resistance levels of NEO in both farm types in the western region. Additional information about the CO pig farm in the western and the northeastern regions are that limited antimicrobial use applied in the farm for approximately the last 6.5 and 3 years, respectively, whereas no antimicrobial use was applied in the DE pig farm in the western and northeastern regions for approximately the last 9 and 5 years, respectively. This additional information would support the results of the lower prevalence value of antimicrobial resistance for a longer period after applying limited or no antimicrobial use in the farms. This agreed with the report [18] of over 11 months required to decrease the proportion of MDR isolates and increased in isolates susceptible to the antimicrobials for pig farms. These results also agreed with a preliminary study of the author (unpublished data) conducted approximately 2 years before this study in the DE pig farm from the western region. That study found a higher proportion of AMX, GEN, KAN, and NEO when compared to the results of this study. Although the overall resistance level of SXT in the DE system was lower than that in the CO system, there was no difference in SXT resistance level in the northeastern region, which was a relative high level from 70% to 80%. This rather high level of resistance to trimethoprim–sulfamethoxazole might be the result of extended-spectrum beta-lactamase (ESBL)-*E. coli* reported in Thailand [7,19,20]. Recently, Gruel et al [21] reported the rate of trimethoprim–sulfamethoxazole resistance occurrence of 72.7% for trimethoprim–sulfamethoxazole resistance in ESBL-*E. coli* versus 13.7% in non-ESBL-*E. coli*, although trimethoprim–sulfamethoxazole was not declared to be used. Gruel et al [21] also reported the rate of ampicillin resistance occurrence at 100.0% for ampicillin resistance in ESBL-*E. coli* versus 14.4% in non-ESBL-*E. coli*, although β-lactams were used by half of the pig farmers. This report would support the high rate of AMX resistance in *E. coli* for the current study, apart from extensive antimicrobial use [5,16,22] in the past. The absence of or very low level of COL and ENR resistance was a positive sign of a very low resistance rate occurring in these two antimicrobials. However, resistance to COL was found in the CO farm located in northeastern Thailand following the report of Khine et al [6]. For discrepancy results between the studied regions, the difference between production systems for NEO resistance was found only in the northeastern region, while the difference between production systems for trimethoprim–sulfamethoxazole resistance was found only in the western region. In the case of NEO resistance, a statistical difference could not be detected, although the prevalence value of the NEO resistance in the DE system (5.45%) was around half of that in the CO system (11.67%). This might be the result of a rather low number of isolates found with resistance (3 and 7). For trimethoprim–sulfamethoxazole resistance, there was a rather high number of isolates found with resistance (35 and 40). Thus, a shorter period after stopping antimicrobial use in the DE pig farm from the northeastern region would be an explanation for the insignificant results. Potential reservoirs of resistant microorganisms in farm environments would also be partly influenced.

The obvious results in the current study showed that the number of antimicrobial-resistant agents in the DE system was lower than (p<0.01) that in the CO system for both studied regions. This result could be the consequence of a high proportion of pigs (approximately 19%) with no antimicrobial resistance and one antimicrobial agent resistance in the DE system, while only 3.6% was found in the CO system. Simultaneously, there was a low proportion of pigs for the MDR with more than 3 antimicrobial agents in the DE system, whereas a high proportion of that existed in the CO system. These results implied the partial success of antimicrobial resistance reduction in pig production by applying the DE system for pig farms. In practice, the main farmers who produce pigs in the DE system have stopped antimicrobial use in their farms. This would be comparable to the successful reduction of antimicrobial resistance by changing from a conventional to an experimentally organic dairy farm [23], in agreement with lower antimicrobial resistance in organic pig farming for commensal *E. coli* [24,25] and *Enterococcus* spp. [25] when compared to conventional pig farming. When pig age was classified into 3 age classes (sow, nursery and starter pig, and grower and finisher pig), it was rather obvious that the grower and finisher pigs played a crucial role in the difference in the number of antimicrobial resistance agents between the pigs in the CO and the DE farms. The highest value of number of antimicrobial resistance agents in all study farms with the highest in the CO farm in the northeastern region indicated a similar trend to the results of earlier reports [5]. In this report, a likely higher antimicrobial-resistant gene found in 3-week-old weaning piglets than their sows and 24 weeks old at the finishing stage, consistent with the higher number of *E. coli*, indicating the diversity of the microbiome which shifted over time during production stages. However, there was a cross-sectional sampling in this study, which made it hard to explain the studied results because of various possible confounding factors, such as weaned piglets housed in a particular pen possibly came from sows with differently carried resistant genes. When comparing the results of the number of antimicrobial resistance agents for the pigs from the DE system in the current study with the longitudinal study in the earlier report [5], there were consistent results. This could be explained by the fact that weaned piglets born from a specific sow were regularly housed in a specific pen without including weaned piglets born from different sows and were then housed in a specific pen for starter, grower, and finisher periods. This would presumably be related to a longitudinal study. Additionally, proportion sampling for testing in accordance with the total number of pigs in each age class would be suitable to get representative results of antimicrobial resistance in each studied farm in term of sampling bias reduction.

Pholwat et al [5] reported that antimicrobial resistance acquisition may occur both from the mother and after weaning, presumably from other sources in the environment. Hence, accumulated microbial agents in litter layers of the deep litter system might provide a greater chance to increase the proportion of antimicrobial-resistant *E. coli*, especially in the case of long-term antimicrobial use in feed, resulting in the accumulation of antimicrobials. It may partly explain the high resistance rates in meat duck reared in deep litter systems [26]. There is not much information available about the deep litter system for pigs, especially related information concerning antimicrobial resistance, even in other livestock animals. One publication, Li et al [27], reported that the different layers of the deep litter system for pigs contained a different group of micro-organisms; aerobic for the upper layers (0 cm to −5 cm), micro-aerobic for of the middle layers (−10 cm to −20 cm) and anaerobic for the bottom layers (below −20 cm depth). They also suggested expanding the aerobic layers of the deep litter system should be applied. Thus, suitable management for deep litter floors might reduce the chance for accumulation of *E. coli*, including antimicrobial-resistant *E. coli*. The report about the natural behaviors of pigs for the pigs’ rooting was induced by bedding mostly comprises rice husks and other materials [28], implying partial expansion of the aerobic layers of the deep litter system, probably resulting in low accumulation of *E. coli* in the farms with the DE system. Concerning possible reasons for the persistence of antimicrobial resistance after the limitation of antimicrobial use in the CO pig farms and even the cessation of antimicrobial use in the DE pig farms would be: i) the maintenance of antimicrobial resistance in bacterial populations [16] through compensatory mutations and plasmid addiction systems from both *E. coli* and other bacteria, and ii) potential reservoir of resistant microorganisms in a farm environment, which re-colonized pigs [19], including added antimicrobial-resistant genes of microorganisms from human working in pig farms or by obtaining them from other sources outside pig farms. Thus, the persistence period of antimicrobial resistance would depend on background and current conditions in each farm. However, based on the study of De Lucia et al [18], it was anticipated that an approximately 15% chance that a decrease of 10% per time point from an initial prevalence ranging from 30% to 50% would be detected as statistically significant. This leads to the implication of using a rather long period to eliminate antimicrobial resistance from a pig farm after stopping antimicrobial use. It was noted that there was the prevalence of 100% (of the total of 25 pigs) antimicrobial resistance found in the DE pig farm from the western region (unpublished data) used in the current study, which found 90.91% (a total of 55 pigs) antimicrobial resistance prevalence, implying 9.09% of antimicrobial-resistant reduction at an approximately 2-year interval without any change of key farm management and policy.

In terms of deep litter system management for pigs, indigenous microorganisms were proven to reduce the foul odor from pig production [29]. Pigs reared in this system were mainly fed fermented feed [30]. From this point of view, there is still a lack of information linked to antimicrobial resistance. Therefore, the appropriate use of microorganisms without antimicrobial resistance should be considered in principle.

For the present study, the researchers did not plan to detect antimicrobial resistance genes (genotypic resistance), although it was an important way to explain the genetic change, mechanism, and transfer, especially for longitudinal study in pig farms and experiments concerning pig farm environments. However, an earlier study [5] showed a correlation between genotypic and phenotypic resistance to *E. coli* from pigs. This would be presumable for the results of the present study as well. Thus, available data obtained from the current study for phenotypic resistance likely illustrates the usefulness of the DE system to alleviate antimicrobial resistance in pig production. To fully elucidate the influences of the DE system on antimicrobial resistance, further study should be performed in terms of larger populations in other farms or regions, as well as detecting antimicrobial resistance genes, especially longitudinal study with genotypic resistance. In addition, there is little information available for deep litter systems for pig production. Thus, other aspects of further study related to deep litter systems would be valuable and useful information.

## CONCLUSION

Comparing the influence of two pig production systems, namely CO and DE systems, on phenotypic antimicrobial resistance by using the disc diffusion method was performed on pig farms located in western and northeastern Thailand. Deep litter system for pigs without antimicrobial use in farms could lower the resistance level of fecal *E. coli* to some studied antimicrobials (GEN, NEO, and SXT), lower the number of antimicrobial-resistant agents, and induce a small proportion of pigs to be free from antimicrobial resistance when compared to the CO system with limited antimicrobial use.

## Figures and Tables

**Table 1:** Farm information and antimicrobial use for each farm in the current study

Production system	Number of sows (total pigs/yr)	Farm location	Antimicrobial use	Antimicrobial classes^1)^
Conventional system (CO)	18 (534)	Western	Limited (treatment only)	β-lactam; Aminoglycosides, Fluoroquinolone
	19 (445)	Northeastern	Limited (treatment only)	β-lactam; Aminoglycosides, Fluoroquinolone
Deep litter system (DE)	41 (534)	Western	No	-
	45 (771)	Northeastern	No	-

1) Antimicrobial agent used in the studied farms for each antimicrobial class: β-lactam = penicillin, amoxicillin; Aminoglycosides = gentamicin, neomycin, streptomycin; Fluoroquinolone = enrofloxacin.

**Table 2:** Percentages of antimicrobial-resistant *Escherichia coli* between the conventional system (CO) and the deep litter system (DE) in the current study

Antimicrobial agents	% Resistant (n)		p-value	% Resistant (n)		p-value	% Resistant (n)		p-value
		
	Western			Northeastern			Overall		
		
	CO (n = 60)	DE (n = 55)		CO (n = 50)	DE (n = 50)		CO (n = 110)	DE (n = 105)	
Amoxicillin	96.67% (58)	89.09% (49)	0.1107	100.00% (50)	98.00% (49)	0.3149	98.18% (108)	93.33% (98)	0.0760
Colistin	0.00% (0)	0.00% (0)	1.0000	4.00% (2)	0.00% (0)	0.1531	1.82% (2)	0.00% (0)	0.1651
Doxycycline	38.33% (23)	50.91% (28)	0.1751	80.00% (40)	66.00% (33)	0.1149	57.27% (63)	58.10% (61)	0.9029
Enrofloxacin	0.00% (0)	0.00% (0)	1.0000	0.00% (0)	2.00% (1)	0.3149	0.00 % (0)	0.95% (1)	0.3049
Gentamicin	20.00% (12)	3.64% (2)	0.0073	28.00% (24)	10.00% (5)	0.0218	23.64% (26)	6.67% (7)	0.0006
Kanamycin	11.67% (7)	1.82% (1)	0.0381	20.00% (10)	26.00% (13)	0.4759	15.45% (17)	13.33% (14)	0.6581
Neomycin	11.67% (7)	5.45% (3)	0.2376	52.00% (26)	26.00% (13)	0.0077	30.00 % (33)	15.24% (16)	0.0099
Trimethoprim-sulfamethoxazole	80.00% (48)	45.45% (25)	0.0001	80.00% (40)	70.00% (35)	0.2482	80.00% (88)	57.14% (60)	0.0003

**Table 3:** Quantity aspect of antimicrobial resistance in *Escherichia coli* isolates from the conventional system (CO) and the deep litter system (DE) of the current study

Items	No. of antimicrobial resistance agents		p-value	No. of antimicrobial resistance agents		p-value	No. of antimicrobial resistance agents		p-value
		
	Western			Northeastern			Overall		
		
	CO	DE		CO	DE		CO	DE	
Sow (n)	11	13		12	15		24	28	
Min-Median-Max	1-2-4	1-2-4	NA	1-3-3	2-3-6	NA	1-2-4	1-3-6	NA
Average	2.09	2.15^ab^	0.8289	2.54^b^	3.13^ab^	0.1353	2.33^b^	2.68^a^	0.3061
Nursery and starter (n)	11	13		24	14		34	27	
Min-Median-Max	2-3-6	2-3-5	NA	2-4-7	2-4-6	NA	2-4-7	2-3-6	NA
Average	3.09	2.69^a^	0.5133	4.57^a^	3.86^a^	0.0978	4.09^a^	3.30^a^	0.0218
Grower and finisher (n)	38	29		14	21		52	50	
Min-Median-Max	1-3-4	0-2-3	NA	3-3-4	0-2-4	NA	1-3-4	0-2-4	NA
Average	2.58	1.55^b^	<0.001	3.14^b^	2.29^b^	<0.01	2.73^b^	1.86^b^	<0.001
Overall age class (n)	60	55		50	50		110	105	
Min-Median-Max	1-2-6	0-2-5	NA	1-3-7	0-3-6	NA	1-3-7	0-2-6	NA
Average	2.58	1.96	<0.01	3.64	2.98	<0.01	3.06	2.45	<0.001

NA, not applicable.

a,b Different superscripts within columns (excluding overall age class item) represent significant differences between age class within each pig farm (p<0.05).

**Table 4:** Ten most common antibiograms of *Escherichia coli* isolates of the conventional system (CO) and the deep litter system (DE) in the current study

Pattern	Profile	Number of resistant antimicrobials	% Isolate (n)					

			CO			DE		
	
			W	NE	W+NE	W	NE	W+NE
1	No resistance	0	0.00 (0)	0.00 (0)	0.0 (0)	9.09 (5)	2.00 (1)	5.71 (6)
2	AMX	1	5.00 (3)	2.00 (1)	3.64 (4)	18.18 (10)	8.00 (4)	13.33 (14)
3	AMX-DOX	2	10.00 (6)	6.00 (3)	8.18 (9)	21.82 (12)	8.00 (4)	15.24 (16)
4	AMX-SXT	2	33.33 (20)	2.00 (1)	19.09 (21)	20.00 (11)	12.00 (6)	16.19 (17)
5	AMX-DOX-SXT	3	15.00 (9)	28.00 (14)	20.91 (23)	20.00 (11)	30.00 (15)	24.76 (26)
6	AMX-GEN-SXT	3	10.00 (6)	2.00 (1)	6.36 (7)	0.00 (0)	0.00 (0)	0.00 (0)
7	AMX-DOX-GEN-SXT	4	6.67 (4)	18.00 (9)	11.82 (13)	0.00 (0)	4.00 (2)	1.90 (2)

W, western Thailand; NE, northeastern Thailand; AMX, amoxicillin; DOX, doxycycline; GEN, gentamicin; SXT, trimethoprim-sulfamethoxazole.
